# Effect of glutamine on the systemic innate immune response in broiler chickens challenged with *Salmonella pullorum*

**DOI:** 10.1186/s12917-023-03836-5

**Published:** 2023-12-16

**Authors:** Qiu jue Wu, Long long Zhu, Rong kai Zhang, Zhong ying Xing, Cong Wang, Jia hui Liao, Nai zhi Hu, Bin yao Cheng, Yan Ma, Yu qin Wang

**Affiliations:** College of Animal Science and Technology, Henan University of Science and Technology, Henan, 471003 China

**Keywords:** Glutamine, Innate immune response, *Salmonella pullorum*, Spleen, Broiler chickens

## Abstract

**Background:**

The objective of this study was to evaluate the effects of glutamine on the growth performance and systemic innate immune response in broiler chickens challenged with *Salmonella pullorum*. A total of 600 one-day-old Arbor Acres broiler chickens were assigned randomly to 6 dietary treatments with 10 replicates for a 21-day feeding experiment. The experimental treatments were as follows: the control treatment (birds fed the basal diet), the Gln1 treatment, and the Gln 2 treatment (birds fed the basal diet supplemented with 0.5%, and 1.0% Glutamine, respectively). At 3 d of age, half of the birds from each treatment were challenged oral gavage with 2.0 × 10^4^ CFU/mL of *S. pullorum* suspension (1.0 mL per bird) or an equivalent amount of sterile saline alone, which served as a control.

**Results:**

The results showed that *S. pullorum* infection had adverse effects on the average daily feed intake, average daily gain, and feed conversion ratio of broiler chickens compared with those of the CON treatment on d 7, decreased the spleen and bursa of fabricius relative weights (except on d 21), serum immunoglobulin A (IgA),immunoglobulin G (IgG), and immunoglobulin M (IgM) concentrations, and spleen melanoma differentiation-associated gene 5 (MDA5) and laboratory of genetics and physiology gene 2 (LGP2) mRNA expression levels, and increased the mRNA expression levels of spleen Nodinitib-1 (NOD1), Toll-like receptors 2,4 (TLR2, TLR4), DNA-dependent activator of IFN-regulatory factors (DAI), mitochondrial antiviral-signaling protein (MAVS), P50, P65, and RelB on d 4, 7, 14, and 21.

Supplementation with Gln improved the relative weights of the spleen and bursa of Fabricius (except on d 21), increased the serum IgA, IgG, and IgM concentrations and the mRNA expression levels of spleen MDA5 and LGP2, and decreased the mRNA expression levels of spleen NOD1, TLR2, TLR4, DAI, MAVS, P50, P65, and RelB of *S. pullorum*-challenged broiler chickens.

**Conclusion:**

These results indicate that Gln might stimulate the systemic innate immune responses of the spleen in broiler chickens challenged with *S. pullorum*.

## Background

*Salmonella*, a Gram-negative bacterium, can initiate humoral and cellular immune responses in multiple organs, such as the liver and spleen, leading to cell death and maturation of antibodies and cytokines, which can cause white diarrhea with a high mortality rate in 2- to 3-week-old chicks, thus causing substantial economic losses in the poultry industry [1, 2]. Recently, a study demonstrated that *Salmonella* infection can induce the innate immune antibody response [3], the cytokine signaling response, and the mRNA expression of immune cell signaling proteins (such as Toll-like receptors 2, 4 (TLR2, TLR4)), which significantly modulate the susceptibility of broiler chicken organs to *Salmonella* [3–5]. Therefore, we focused on a viable additive that would provide modest protection for poultry at high risk of being infected with *Salmonella*, which is likely to become increasingly important in the future.

Glutamine (Gln) is assumed to be a conditionally indispensable amino acid, especially in challenging periods, is an important energy fuel for immune cells, and usually appears on the list of “immunonutrients” [6]. There is evidence from animal and clinical studies that Gln possesses immunoenhancing properties by influencing the amount and function of T lymphocytes, as well as the production of some cytokines by T cells, tumor growth, and activation of killer cells by lymphocytes [7, 8]. Extensive in vitro and in vivo studies on the anti-inflammatory and immune properties of Gln have shown that Gln significantly decreases the percentage of heterophils, the ratio of heterophils to lymphocytes, (interleukin (IL))-8 production, and p38 mitogen-activated protein kinase protein levels [9, 10]. Moreover, addition of Gln could also increase the percentage of lymphocytes, the proliferation of T and B lymphocytes in peripheral blood, the inhibitor of nuclear factor kappa B α content, and the nuclear transcription factor kappa B p50 and p65 expression in injured rats and stress-treated broiler chickens or piglets [9]. Some studies showed that Gln supplementation reduced the expression of TLR2, TLR4, myeloid differentiation factor (MyD) 88, Tumor necrosis factor-alpha (TNF-α) receptor associated factor 6, and caspase-3 and significantly protected against organ injury under stress conditions [11–13]. Taken together, these results highlight the physiological importance of Gln, which contributes to attenuating the body’s inflammatory response by counteracting the nuclear factor kappa-B (NF-κB) and TLR pathway activation. Therefore, there is a deficiency in the arterial supply of Gln to the systemic innate immune response in an attempt to help body growth demand. However, previous studies have not yet explored the systemic innate immune mechanisms involved in Gln protection against *Salmonella* in poultry. The purpose of this study was to test the innate immune effects of Gln on the spleen against *Salmonella* infection in broiler chickens.

## Methods

### Salmonella pullorum, Gln

The *S. pullorum* serotype used in this study was purchased from the China Veterinary Culture Collection Center (CVCC 3377 (12/10/2018-11/10/2023), Beijing, China). Cultures of selected *S. pullorum* were grown anaerobically on Brilliant Green Agar at 37 °C for 24 h, washed, and diluted to 2.0 × 10^4^ colony-forming unit (CFU)/mL in sterile normal saline. A colony count on a standard plate confirmed the viable cell count of the infected *S. pullorum* strain.

Glutamine (pharmaceutical grade: 99 % purity) was purchased from Henan Honda Biological Medicine Co., Ltd., China, and was used in the basal feed.

### Broiler chickens, management, experimental diets and experimental design

A total of 600 one-day-old Arbor Acres (AA*,* 51.28 ± 0.07 g) weighed broiler chickens were obtained from a commercial hatchery (Broiler Breeding Farm of Shiqiao Complex Productive Co., China), and were randomly assigned to 6 dietary treatments with 10 replicates of 10 birds (half male and half female) per replicate for a 21-day feeding experiment.

This experiment used a 2 × 3 factorial design. There were 6 dietary treatments: the control treatment (birds fed the basal diet, CON) and two Gln treatments (birds fed the basal diet supplemented with 0.5 %, and 1.0 % Gln, respectively). At 3 d of age, half of the birds from each treatment were then given oral gavage with 2.0 × 10^4^ CFU/mL of *S. pullorum* suspension (1.0 mL per bird) or an equivalent amount of sterile saline alone, which served as a control. The doses and routes of *S. pullorum* administration referred to the previous study [5]. Each replicate (10 birds) was considered the experimental unit for dietary treatment and challenge status (oral gavage with *S. pullorum* suspension or saline).

All the broiler chickens were weighed and placed in metal cages (150 cm × 100 cm × 60 cm) in an environmentally controlled room; the broiler chickens were exposed to ambient temperatures descending from 34 °C to 22 °C ± 1℃ for 21 days. Plastic separators were used to prevent horizontal contamination. There were twenty-three hours of light on days 1 to 7 and 18 hours on days 8 to 21. The broiler chickens were fed ad libitum, and their feed intake and body weight were measured. The average daily feed intake (ADFI), average daily gain (ADG), and feed conversion ratio (FCR) were calculated. The broiler chickens were fed standard starter feed (corn–soybean meal) in mash. The diet was formulated to meet or exceed the nutrient requirements for broiler chickens as recommended by the National Research Council (NRC, 2018) (Table 1). The experimental animal, design and process were authorized by the guidelines of the Institutional Animal Care and Use Committee of Henan University of Science and Technology (approve number: 052-2023).

**Table 1:** Composition of diets in the experiment for broiler chickens being d 1to d 21 (DM basis)

Items, g/kg	d 1to d 21	Calculated nutrients	d 1to d 21
Corn	428	DM,%	89.13
Soybean meal (43%, crude protein)	365	CP,g/kg	222
Wheat	130	Ca,g/kg	9.7
Soybean oil	17	AP,g/kg	4.7
Corn gluten meal	20	Lys,g/kg	13.8
Na chloride	2.3	Met,g/kg	6.0
Dicalcium phosphate	15	Met + cys,g/kg	8.1
Na bicarbonate	2.4		
Ca carbonate	10.8		
DL-Met	2.7		
L-Lys	2.2		
Premix^a^	2		
Multi-enzyme	0.3		
Phytase	0.3		
Mannan oligosaccharides	2		
Total	1, 000		

^a^Per kilogram premix of diet: vitamin A, 10,000 IU; vitamin D_3_, 3,000 IU; vitamin E, 30 IU; vitamin K3, 1.3 mg; vitamin B1, 2.2 mg; vitamin B2, 8 mg; vitamin B3, 40 mg; choline chloride, 600 mg; D-pantothenate, 10 mg; vitamin B6, 4 mg; biotin, 0.04 mg; folic acid, 1 mg; vitamin B_12_, 0.013 mg; Fe, 80 mg; Cu, 8 mg; Mn, 110 mg; Zn, 65 mg; iodine, 1.1 mg; Se, 0.3 mg

### Sample collection and procedures

On d 1, 7, 14, and 21, each broiler chicken was weighed after an overnight fast. In addition to weighing feed and feed residues, the ADFI and FCR were also determined; on days 4, 7, 14, and 21, the chickens fasted for 12 hours before sampling. We randomly selected 10 broiler chickens from each treatment and immediately dislocated their cervical vertebrae; the remaining animals were released to chicken house and raised together with other non-experimental animals. In order to collect serum samples (5.0 mL), blood was drawn from the caudal veins of broiler from each replicate with momentary automatic venous blood sampler, centrifuged for 15 minutes at 1,800 g, and then stored at -80°C for future immunoglobulin (Ig) tests. In the following step, the broiler chicken were euthanized, using electrocution followed by cervical dislocation, and carcasses were aseptically opened, and the spleen and bursa of fabricius samples were surgically removed to an ice-cold plate and weighed to calculate the immune organ indices using the following formula: immune organ indices (g/kg) = organ weight (g) / live body weight (kg). Each chicken's spleen sample was dissected into two subsamples, one of which was stored quickly at -80 °C in order to assess the quality of the RNA.

### Detection of the serum immunoglobulin populations

Enzyme-linked immunosorbent assays (ELISAs) were performed using Bio-ELISA Toxo- immunoglobulin (Ig) A, IgM, and IgG kits (BlueGene, Shanghai, People’s Republic of China) according to the manufacturer’s instructions. The levels of IgA, IgG and IgM were examined using a commercial immunoassay kit with goat anti-chicken IgG and IgM (BlueGene, Shanghai, People’s Republic of China). There was a test limit of 0.1 g/mL for the assay.

### Spleen sample RNA extraction and qRT-PCR analysis

TRIzol reagent (Invitrogen Trading (Shanghai) Co., Ltd., China) was used to extract the total RNA from the spleen samples (2 grams). According to the manufacturer's recommendations, the tissues were ground using a homogenizer. Reverse transcription was performed by using the High-Capacity cDNA Reverse Transcription Kit (Applied Biosystems, Carlsbad, CA), according to the manufacturer’s protocol. The primer sequences for glyceraldehyde-3-phosphate dehydrogenase (GAPDH), Nodinitib-1 (NOD1), TLR2, TLR4, melanoma differentiation-associated gene 5 (MDA5), DNA-dependent activator of IFN-regulatory factors (DAI), laboratory of genetics and physiology gene 2 (LGP2), mitochondrial antiviral-signaling protein (MAVS), P50, P65, and RelB were designed with the Primer Premier Software (PREMIER Biosoft International, USA), according to the known chicken sequences, as provided in Table 2. Each reaction was run in duplicate using the SYBR® Green RT-PCR Kit and SYBR® Green Supermix as the qRT-PCR master mix. The PCR amplification parameters were: 2 min at 50 °C, 20 min at 95 °C, and 40 cycles for 10 seconds. The absorbance at 260 nm and 280 nm was calculated. In this experiment, the cycle threshold values from the qRT-PCR were recorded and analyzed with the help of the 7500 Real-Time PCR software (Applied Biosystems).The relative fold-change from different parts of the spleen was calculated by the 2−ΔΔct method, normalized to the gene-specific efficiencies of the abovementioned index, which is a measure of the gene-specific efficiencies [14].

**Table 2:** Gene specific sequences primers used in real-time quantitative PCR

Target genes	Primers and probes sequence (5’ → 3’)
NOD1	AGGAGCTCTCATCAGCGAAGATCT
	GCAGCGTCAGCAGAAGACCATT
TLR2	CCTGGTGTTCGTGTTCATCCTCAT
	AGTTGGAGTCCTTCTCACTGTAGG
TLR4	ACGGATGGCTTTGGTTGGGATTA
	GATGTAGCTATCTGGTGCTTGGAAT
MDA5	CAGCGAGTTGCCCTAGCCTCA
	AACACCTCCCTTCGCCCGTCT
DAI	CACGCGGTTGGTGCTATCATTG
	ATACTGCCTTGTGCCTCGAACTG
LGP2	CCAGAATGAGCAGCAGGAC
	AATGTTGCACTCAGGGATGT
MAVS	CCTGACTCAAACAAGGGAAG
	AATCAGAGCGATGCCAACAG
P50	CAGCCAACTGGGAGGTGTAT
	GCTGCTTGGAGCTTTCATTC
P65	GTGTGAAGAAACGGGAACTG
	GGCACGGTTGTCATAGATGG
RelB	GTGCTGTCTGAGCCCATCTTCG
	TACGGCGGCGTTCGG
GAPDH	GTGGTGGCCATCAATGATCC
	ACTTGTGATCAATGGGCACG

### Statistical analysis

Statistical analyses of all the data were performed by two-way ANOVA using the GLM procedure of statistical software SPSS version 21.0 (SPSS Inc., Chicago, IL, USA, 2012) as a 2 × 3 factorial arrangement with the diet and *S. pullorum* as the main effects. One-way ANOVA using Duncan's multiple range tests was used when the interaction between the main effects was less than 0.10. A difference between the means at *P*< 0.05 was considered statistically significant. The treatment means for all the data are presented with their pooled SEM.

## Results

### Growth performance

The effects of the dietary Gln supplementation on the growth performance of broiler chickens infected with *S. pullorum* are displayed in Table 3. Among the *S. pullorum*-unchallenged treatments, the Gln treatments increased the ADG and decreased the ADFI and FCR compared with the CON treatment, but there were no differences in the growth performance between the 0.5 % Gln and 1.0 % Gln treatments on d 14 and 21. *S. pullorum* infection had a negative effect on the daily feed intake on d 14 and 21. Compared with the *S. pullorum* treatment, the Gln treatments significantly improved the average daily gain and feed conversion ratio of the broiler chickens on d 7. On day 7, the ADG increased and the ADFI and FCR decreased in *S. pullorum*-challenged broiler chickens. Furthermore, there were stress–diet interactions on D7 in terms of ADG, ADFI, and FCR for *S. pullorum*.

**Table 3:** Effect of dietary Gln on growth performance and relative weight of immune organ of broiler chickens infected with *Salmonella pullorum*

Items^b^	CON^a^	Gln (%)^a^		Scc^a^	Gln^a^ (%) + *S. pullorum*		Mean	SEM	*P*-values^c^		
		0.5	1.0		0.5	1.0			Stress	Diet	Interaction
d 1 to 4											
ADFI(g/bird/d)	20.17	20.63	20.69	20.03	20.54	20.12	20.36	0.15	0.013	0.298	0.821
ADG(g/bird/d)	15.40	15.87	15.81	14.84	15.33	15.48	15.46	0.19	0.011	0.301	0.103
FCR(g/g)	1.31	1.30	1.31	1.35	1.34	1.30	1.32	0.05	0.008	0.225	0.321
Spleen(g/kg)	9.18	9.74	9.76	6.50	8.90	9.32	8.91	1.68	< 0.001	0.001	0.001
Bursa of fabricius(g/kg)	10.77	11.25	11.29	9.16	9.90	10.61	10.49	1.89	0.005	0.015	0.023
d 5 to 7											
ADFI(g/bird/d)	27.48	27.05	26.90	28.29	27.64	27.24	27.43	0.22	0.042	0.037	0.013
ADG(g/bird/d)	19.77	20.81	20.85	18.86	19.33	19.74	19.89	0.12	0.039	0.026	0.032
FCR(g/g)	1.39	1.30	1.29	1.50	1.43	1.38	1.38	0.04	0.024	0.041	0.037
Spleen(g/kg)	11.36	14.19	14.18	7.27	10.86	10.90	11.46	1.86	0.003	0.012	0.003
Bursa of fabricius(g/kg)	13.12	15.94	16.07	10.06	12.81	13.42	13.57	2.31	0.005	0.011	0.005
d 8 to 14											
ADFI(g/bird/d)	31.71	30.82	30.78	32.73	32.33	30.20	31.43	0.85	0.128	0.239	0.765
ADG(g/bird/d)	21.57	22.01	21.99	21.39	21.22	21.57	21.63	0.72	0.051	0.312	0.684
FCR(g/g)	1.47	1.40	1.40	1.53	1.50	1.45	1.46	0.07	0.112	0.120	0.274
Spleen(g/kg)	16.32	20.42	20.56	13.25a	17.93b	17.28	17.63	1.73	0.011	0.047	0.019
Bursa of fabricius(g/kg)	14.54	17.68	17.79	11.04a	14.10b	14.44	14.93	1.18	0.019	0.031	0.047
d 15 to 21											
ADFI(g/bird/d)	40.65	45.33	45.17	38.50	44.54	43.52	42.97	0.25	0.215	0.367	0.725
ADG(g/bird/d)	24.64	27.81	27.88	21.88	26.20	25.90	25.72	0.54	0.321	0.128	0.864
FCR(g/g)	1.65	1.63	1.62	1.76	1.70	1.68	1.67	0.05	0.147	0.231	0.647
Spleen(g/kg)	21.52	23.69	23.70	18.30	20.91	21.35	21.58	1.21	0.027	0.041	0.049
Bursa of fabricius(g/kg)	15.71	16.94	17.03	15.50	16.50	16.13	16.30	1.12	0.057	0.053	0.067

^a^Glutamine per kg standard diet feed: Con, 0% Gln, noninfect control treatment; 0.5%Gln = basal diet supplemented with 0.5% Gln; 1.0%Gln = basal diet supplemented with 1.0% Gln. Scc = *Salmonella pullorum* infect control treatment received the basal diet

^b^*ADG* Average daily weight gain, *ADFI* Average daily feed intake, *FCR* Feed conversion ratio

^c^The *P*-values represent the main effect of the diet, the main effect of *Salmonella pullorum* challenge, and the interaction between the dietary treatments and *Salmonella pullorum* challenge

### The relative weights of the immune organs

The effects of the dietary Gln supplementation on the relative weights of the immune organs of broiler chickens infected with *S. pullorum* are displayed in Table 3. On d 4, 7, 14, and 21, among the *S. pullorum*-unchallenged treatments, the Gln treatments increased the relative weights of the spleen and bursa of fabricius compared with the CON treatment, but there were no differences in the relative weights of the spleen and bursa of fabricius between the 0.5 % Gln and 1.0 % Gln treatments. *S. pullorum* infection decreased the relative weights of the spleen and bursa of fabricius compared with those of the CON treatment (Table 3). Compared with the *S. pullorum* treatment, the Gln treatments had increased relative weights of the spleen and bursa of fabricius of *S. pullorum*-challenged broiler chickens, but there were no differences in the relative weights of the spleen and bursa of fabricius between the CON and Gln plus *S. pullorum*-challenged treatments. Additionally, there were *S. pullorum* stress × diet interactions on the relative weights of the spleen and bursa of fabricius.

### IgA, IgG and IgM concentrations in the serum

As shown in Table 4, the Gln treatments increased the IgA, IgG, and IgM levels in the serum, but there were no differences in the immunoglobulin concentrations between the 0.5 % Gln and 1.0 % Gln treatments. On days 4, 7, 14 and 21, the *S. pullorum* infection decreased the serum concentrations of the IgA, IgG, and IgM compared to the CON treatment. The Gln treatments increased the concentrations of the IgA, IgG and IgM in the serum of the *S. pullorum*-challenged broiler chickens compared with those of the *S. pullorum*-infection treatment on d 4, 7, 14, and 21. However, there were no differences in the concentrations of serum IgA, IgM, and IgG between the CON and Gln plus *S. pullorum*-challenged treatments on d 4, 7, 14, and 21. Additionally, there were *S. pullorum* stress × diet interactions on the concentrations of IgA, IgG, and IgM in the serum.

**Table 4:** Effect of dietary Gln on the antibodies levels in the serum of broiler chickens infected with *Salmonella pullorum*

Items^b^	CON^a^	Gln (%)^a^		Scc^a^	Gln^a^ (%) + *S. pullorum*		Mean	SEM	*P*-values^c^		
		0.5	1.0		0.5	1.0			Stress	Diet	Interaction
d 4											
IgG(mg/mL)	2.01	2.23	2.30	1.74	1.92	1.97	2.03	0.12	0.023	0.025	< 0.001
IgM(μg/mL)	600.6	737.0	735.2	451.4	584.6	589.9	516.5	20.9	0.035	0.051	0.042
IgA(mg/mL)	1.24	1.41	1.46	1.07	1.20	1.21	1.09	0.06	0.013	0.031	0.011
d 7											
IgG(mg/mL)	3.58	4.70	4.68	2.33	3.44	3.55	3.71	0.17	0.038	0.022	0.012
IgM(μg/mL)	642.3	779.6	783.6	479.5	628.1	628.7	656.9	13.9	0.018	0.040	0.045
IgA(mg/mL)	1.35	1.46	1.50	1.16	1.26	1.32	1.34	0.07	0.029	0.021	0.044
d 14											
IgG(mg/mL)	3.95	4.62	4.67	3.13	3.86	3.93	4.02	0.15	0.013	0.037	0.018
IgM(μg/mL)	501.4	596.1	595.9	397.1	484.1	486.3	510.2	11.3	0.031	0.021	0.031
IgA(mg/mL)	1.40	1.54	1.55	1.21	1.34	1.39	1.41	0.05	0.017	0.089	0.044
d 21											
IgG(mg/mL)	4.44	4.82	4.81	3.97	4.30	4.41	4.46	0.17	0.037	0.041	< 0.001
IgM(μg/mL)	578.8	611.1	615.3	413.5	565.5	581.1	560.9	15.1	0.029	0.039	0.037
IgA(mg/mL)	1.55	1.74	1.73	1.29	1.46	1.47	1.54	0.07	0.028	0.037	0.012

^a^Glutamine per kg standard diet feed: Con, 0% Gln, noninfect control treatment; 0.5%Gln = basal diet supplemented with 0.5% Gln; 1.0%Gln = basal diet supplemented with 1.0% Gln. Scc = *Salmonella pullorum* infect control treatment received the basal diet

^b^*IgG* Immunoglobulin G, *IgM* Immunoglobulin M, *IgA* Immunoglobulin A

^c^The *P*-values represent the main effect of the diet, the main effect of *Salmonella pullorum* challenge, and the interaction between the dietary treatments and *Salmonella pullorum* challenge

mRNA Expression levels of the main pattern recognition receptors in the spleen

As shown in Table 5, the dietary Gln influenced the main pattern recognition receptors in the innate immune-related signaling pathways and the regulation of broiler chickens infected with *S. pullorum*. Among the *S. pullorum*-unchallenged treatments, the Gln treatments decreased the NOD1, TLR2, TLR4, DAI, and MAVS mRNA expression levels, and increased the MDA5 and LGP2 mRNA expression levels in the spleen compared with the CON treatment, but there were no differences in these gene expression levels of the spleen between the 0.5 % Gln and 1.0 % Gln treatments on d 4, 7, 14, and 21.

**Table 5:** Effect of dietary Gln on main pattern recognition receptors in innate immune related signaling pathways and regulation of broiler chickens infected with *Salmonella pullorum*

Items^b^	CON^a^	Gln (%)^a^		Scc^a^	Gln^a^ (%) + *S. pullorum*		Mean	SEM	*P*-values^c^		
		0.5	1.0		0.5	1.0			Stress	Diet	Interaction
d 4											
NOD1	0.99	0.75	0.76	1.21	1.07	1.00	0.96	0.14	0.013	0.029	0.021
TLR2	0.78	0.50	0.47	1.20	0.93	0.80	0.78	0.10	0.011	0.031	0.013
TLR4	0.51	0.30	0.28	0.81	0.60	0.50	0.50	0.15	0.008	0.025	0.021
MDA5	1.11	0.85	0.88	0.85	1.10	1.12	0.99	0.16	< 0.001	0.001	0.011
DAI	1.00	0.37	0.38	1.88	1.14	1.10	0.99	0.12	0.005	0.015	0.023
LGP2	1.07	0.74	0.73	0.77	0.99	1.04	0.89	0.14	0.017	0.034	0.033
MAVS	0.27	0.10	0.08	0.77	0.37	0.30	0.32	0.12	0.024	0.017	0.037
d 7											
NOD1	1.08	0.79	0.78	1.49	1.11	1.05	1.05	0.15	0.015	0.021	0.012
TLR2	0.84	0.58	0.59	1.31	1.04	0.96	0.89	0.13	0.017	0.027	0.014
TLR4	0.57	0.38	0.39	0.90	0.68	0.59	0.59	0.12	0.014	0.019	0.027
MDA5	1.20	0.92	0.97	0.91	1.13	1.19	1.05	0.14	0.021	0.041	0.020
DAI	1.07	0.42	0.41	1.97	1.21	1.17	1.04	0.17	0.019	0.021	0.027
LGP2	1.11	0.81	0.80	0.81	1.06	1.10	7.15	0.15	0.030	0.014	0.017
MAVS	0.28	0.12	0.11	0.81	0.39	0.32	0.34	0.16	0.023	0.009	0.014
d 14											
NOD1	1.19	0.82	0.84	1.58	1.21	1.20	1.14	0.11	0.004	0.011	0.014
TLR2	0.87	0.63	0.60	1.39	1.10	1.04	0.94	0.12	0.041	0.031	0.043
TLR4	0.61	0.41	0.46	0.99	0.74	0.65	0.64	0.17	0.023	0.047	0.027
MDA5	1.28	0.99	0.99	0.97	1.24	1.28	1.13	0.13	0.029	0.035	0.040
DAI	1.12	0.50	0.51	2.04	1.30	1.24	0.92	0.18	0.030	0.024	0.017
LGP2	1.19	0.87	0.89	0.86	1.09	1.16	1.01	0.16	0.043	0.028	0.027
MAVS	0.31	0.19	0.20	0.88	0.42	0.37	0.40	0.15	0.018	0.018	0.025
d 21											
NOD1	1.27	0.86	0.87	1.76	1.33	1.28	1.23	0.14	0.017	0.029	0.019
TLR2	0.93	0.69	0.67	1.43	1.17	1.18	1.01	0.10	0.028	0.009	0.041
TLR4	0.69	0.47	0.50	1.05	0.78	0.71	0.70	0.17	0.020	0.018	0.011
MDA5	1.54	1.04	1.09	1.01	1.49	1.42	1.26	0.16	0.028	0.041	0.018
DAI	1.20	0.58	0.56	2.18	1.55	1.30	1.23	0.18	0.018	0.034	0.017
LGP2	1.28	0.93	0.84	0.82	1.20	1.22	1.05	0.12	0.008	0.042	0.027
MAVS	0.37	0.24	0.22	0.94	0.50	0.43	0.45	0.14	0.019	0.027	0.024

^a^Glutamine per kg standard diet feed: Con, 0% Gln, noninfect control treatment; 0.5%Gln = basal diet supplemented with 0.5% Gln; 1.0%Gln = basal diet supplemented with 1.0% Gln. Scc = *Salmonella pullorum* infect control treatment received the basal diet

^b^*NOD1* Nodinitib-1, *TLR2* Toll-like receptors 2, *TLR4* Toll-like receptors 2, *MDA5* Melanoma differentiation-associated gene, *DAI* DNA-dependent activator of IFN-regulatory factors, *LGP2* Laboratory of genetics and physiology gene 2, *MAVS* Mitochondrial antiviral-signaling protein

^c^The *P*-values represent the main effect of the diet, the main effect of *Salmonella pullorum* challenge, and the interaction between the dietary treatments and *Salmonella pullorum* challenge

Compared with those of the CON treatment, *S. pullorum* infection increased the spleen NOD1, TLR2, TLR4, DAI, and MAVS mRNA expression levels, and decreased the spleen MDA5 and LGP2 mRNA expression levels on d 4, 7, 14, and 21, However, the Gln treatments could decrease the spleen NOD1, TLR2, TLR4, DAI, and MAVS mRNA expression levels and increase the spleen MDA5 and LGP2 mRNA expression levels in the *S. pullorum*-challenged broiler chickens treatment on d 4, 7, 14, and 21; and there were no differences in the spleen NOD1, TLR2, TLR4, DAI, LGP2, MDA5, and MAVS between the CON and Gln treatments plus *S. pullorum*-challenged. Additionally, there were *S. pullorum* stress × diet interactions on the mRNA expression levels of the spleen NOD1, TLR2, TLR4, DAI, and MAVS.

### mRNA Expression levels of the transcription factors in the spleen

As shown in Table 6, the dietary Gln affected the transcription factors that relate to the innate immune-related signaling pathways in broiler chickens infected with *S. pullorum*. Among the *S. pullorum*-unchallenged treatments, the Gln treatments decreased the mRNA expression levels of P50, P65, and RelB in the spleen compared with the CON treatment, but the treatments with 0.5 % Gln or 1.0 % Gln showed no differences in the transcription factors mRNA expression levels in spleen on day 4, 7, 14, and 21. Compared with the CON treatment, the *S. pullorum*-infection treatment showed increased mRNA expression levels of P50, P65, and RelB in the spleen on d 4, 7, 14, and 21. Compared with the *S. pullorum*-infected treatment, the spleen mRNA expression levels of P50, P65, and RelB in the Gln treatments plus *S. pullorum*-challenged broiler chickens were lower than those in the *S. pullorum*-infected treatment; there were no differences in the mRNA expression levels of P50 and P65, and RelB between the CON and Gln treatments plus *S. pullorum*-challenged on d 4, 7, 14, and 21. Additionally, there were *S. pullorum* stress × diet interactions on the mRNA expression levels of P50, P65, and RelB.

**Table 6:** Effect of dietary Gln on the transcriptional factors in innate immune related signaling pathways and regulation of broiler chickens infected with *Salmonella pullorum*

Items^b^	CON^a^	Gln (%)^a^		Scc^a^	Gln^a^ (%) + *S. pullorum*		Mean	SEM	*P*-values^b^		
		0.5	1.0		0.5	1.0			Stress	Diet	Interaction
d 4											
P50	0.71	0.31	0.30	1.69	1.00	0.91	0.82	0.12	0.019	0.041	0.014
P65	1.01	0.28	0.27	2.25	1.10	1.06	1.16	0.17	0.031	0.045	0.011
RelB	0.99	0.17	0.15	2.28	1.13	1.04	0.96	0.15	0.035	0.021	0.034
d 7											
P50	0.83	0.31	0.32	1.88	1.08	0.96	0.90	0.13	0.025	0.027	0.017
P65	1.10	0.29	0.28	2.42	1.22	1.18	1.08	0.17	0.017	0.040	0.030
RelB	1.02	0.17	0.16	2.42	1.20	1.11	1.01	0.11	0.033	0.029	0.027
d 14											
P50	0.91	0.33	0.33	2.07	1.17	1.04	0.80	0.12	0.032	0.026	0.020
P65	1.18	0.31	0.30	2.51	1.28	1.22	1.13	0.14	0.031	0.045	0.027
RelB	1.10	0.19	0.21	2.57	1.27	1.16	1.08	0.15	0.028	0.023	0.012
d 21											
P50	0.97	0.36	0.39	2.16	1.24	1.12	1.04	0.16	0.023	0.023	0.031
P65	1.25	0.34	0.33	2.78	1.36	1.28	1.22	0.14	0.019	0.021	0.0415
RelB	1.15	0.22	0.24	2.62	1.34	1.22	1.13	0.13	0.034	0.028	0.029

^a^Glutamine per kg standard diet feed: Con, 0% Gln, noninfect control treatment; 0.5%Gln = basal diet supplemented with 0.5% Gln; 1.0%Gln = basal diet supplemented with 1.0% Gln. Scc = *Salmonella pullorum* infect control treatment received the basal diet

^b^The *P*-values represent the main effect of the diet, the main effect of *Salmonella pullorum* challenge, and the interaction between the dietary treatments and *Salmonella pullorum* challenge

## Discussion

There is a significant negative effect on broiler chicken growth performance, intestinal flora colonization, and gut health caused by *Salmonella* [5, 15]. The present study found that *Salmonella* decreased the growth performance of broiler chickens on d 7 and had no effect on it during other experimental periods. Infections could be prevented, however, by activating the immune response system. Moreover, *S. pullorum* infections in broiler chickens are extremely serious in their first 7 days of life, according to previous studies [13, 16]. In young birds, *S. pullorum* can colonize the gut and cause systemic or septicemic diseases and reduces growth. There was a slight difference between the results of our experiment and those of others [17], which may be a result of the age of the chicks at challenge, strain variations, challenge dosages, and animal management [5].

However, the Gln supplementation improved the ADG, ADFI, and FCR in the *S. pullorum*-challenged broiler chickens on day 7, suggesting that Gln might exert a protective effect, which was similar to the result of Fasina et al. [18]. These improvements are probably attributed to improved digestive system structure and function, intestinal enzyme activity, immunity and antioxidant capacity [19–21]. Gln, a critical gut-trophic nutrient, is a major metabolic fuel for enterocytes and has a positive impact on gain performance in weaned pigs [22], broiler chickens [23], and red drums [24]. According to these studies, Gln may improve the growth performance of broiler chickens under stress conditions, which could be attributed to its growth-enhancing properties and digestion of nutrients, and improvement in apparent nitrogen retention, all occurring during the process of developing the digestive organs. On the other hand, it has been reported that Gln did not improve broiler chicken ADG during stress situations [25]. The difference in results could be directly related to the concentration of Gln, the rearing period [25], as well as the levels and sources of stress.

The spleen and bursa of fabricius are the central and peripheral immune organs of broiler chickens; in addition to producing B and T lymphocytes, they contain a large number of lymphocytes [26]. The size of these immune organs can indicate poultry's immune status [27]. In our study, we observed similar results to those observed by Zuamí et al. [28]; *Salmonella* enterica subsp. enterica serovar typhimurium inoculated into broiler chickens may cause stress. However, earlier studies showed that *Salmonella* enteritidisinfection had no significant effect on immune organ indices in broiler chickens [29, 30]. Broiler chickens are known to suffer from immunosuppression because of the variation in the weights of their immune organs [23]. Moreover, spleen and bursa index were significantly decreased by *S. pullorum* infection in the period 1-4 days of age, although the challenge was done the 3rd day of age, the result may be related to the acute stress reaction.

In our present experimental results, Gln increased the relative weights of the spleen and bursa of fabricius, findings that were similar to those of a previous study by Szabo et al. [31]. In these studies, Gln was found to be effective in counteracting *Salmonella* infection and improving the immune organ indices of broiler chickens, which could facilitate the colonization of beneficial bacteria within the gut. Consequently, broiler chicken growth performance is improved due to improved intestinal health. These effects on the immune organs of broiler chickens could be attributed to the beneficial effect of Gln; T and B lymphocyte development and maturation, as well as the diversification of specific antibodies, may ultimately be responsible [32]. Based on a total, differential, and proliferating peripheral blood T and B lymphocyte count in the broiler chickens in our study, these results were confirmed.

Antibodies are major glycoprotein molecules produced by B lymphocytes, which are also called immunoglobulin. The immune system relies on them to combat bacterial and viral infections. Thus, we investigated the effects of *Salmonella* challenge on the Igs in the serum of the broiler chickens. Our present results verified that *Salmonella* broiler chickens experienced decreased concentrations of serum IgA, IgG, and IgM, especially for the serum immunoglobulin of broilers on d 4. The result shows the view that the Igs are clearly involved in the development of the serum immune response to *Salmonella* and are critical for protecting lymphocytes against toxins, viruses, and bacterial infections [33, 34]. Moreover, the rapid changes in the immunoglobulin content of animal body in a short time could also lead to acute stress reaction. In the present study, we found that Gln could increase the Ig concentrations (IgA, IgG, and IgM) in the serum. The mechanism of Gln's immunity-protecting properties may be partly due to the peripheral immune cell depletion [35]. To gain a thorough understanding of Gln's potential applications in feeding practices, additional studies are needed.

Nodinitib-1, TLR2, TLR4, DAI, LGP2, MDA5, and MAVS are important innate immune receptors that are directly or indirectly activated by inflammatory factors, such as IL-6, IL-12, TNFα, IFNα, and IFNβ. Some studies have demonstrated that increased mRNA expression levels of NOD1, TLR 2, and TLR 4 are associated with proinflammatory responses induced by stress in animal models, providing a novel link between innate immunity and stress-induced inflammation [36, 37]. DAI, a cytosolic DNA sensor, can also activate NF-κB, leading to inflammatory cytokine activation [38]. MAVS, an adaptor protein, may mediate the immunoregulation signaling pathway [39] and can trigger the activation of type I IFN [40] and NF-κB [41] responses with the consequent production of inflammatory cytokines. In the present study, the expression levels of NOD1, TLR2, TLR4, DAI, and MAVS were increased in spleen tissues in the *Salmonella*-infected broiler chickens, suggesting, that the chicken molecule like NOD1, TLR2, TLR4, DAI, and MAVS are involved in the regulation of intracellular inflammatory immune responses by modulating its differentiation [42]. Our results were similar to those observed in the above mentioned experimental results, which the potential mechanisms may be due to the result of strong stress. Moreover, these results also support the hypothesis that the NOD1, TLR2, TLR4, DAI, and MAVS signaling pathways are the important regulators of monocyte activation by mediating the release of interleukins during *S. pullorum* infection in vivo. On the other hand, the variation characters of our results are different at various time intervals, which may be related to the stress intensity, stress duration, and the age of broilers.

Moreover, MDA5 and LGP2 are cytoplasmic proteins, members of the RIG-I-like receptor family, that are positive regulators of viral recognition and subsequent antiviral responses and are involved in the recognition of virus-specific components by innate immune cells (such as TLRs and NOD-like receptors) [43, 44]. In the present study, the expression levels of the spleen MDA5 and LGP2 were decreased, and the TLR2 and TLR4 expression levels correlated with pathological injury in splenic tissue, suggesting that MDA5 and LGP2 may play important roles in the physiological stress response of splenic tissue in the *S. pullorum*-infected broiler chickens.

Glutamine is considered a conditionally indispensable amino acid in metabolic stress situations and plays an important role in improving various functions, as a source of cellular and immunologic energy, in maintaining the integrity and function of various organs (such as intestine, liver, and spleen), and in tissue repair [45]. Studies suggest that Gln inhibits inflamed intestines, livers, and spleens by modulating proinflammatory cytokines and antigens via regulation of the TLR and NOD signaling pathways. There is evidence that *S. pullorum* stimulates lymphocytes to produce proinflammatory cytokines and antigens, thereby contributing to inflammation [46]. Therefore, in our current experiment, Gln supplementation reduced the mRNA expression levels of the spleen NOD1, TLR2, TLR4, DAI, and MAVS and increased the mRNA expression levels of the spleen MDA5 and LGP2 on d 4, 7, 14, and 21 following the *S. pullorum* challenge. Based on these results, the main source of energy for spleen cells appeared to be the dietary Gln; in addition to stimulating nucleic acid synthesis, it may also provide energy to mononuclear cells, such as T lymphocytes. In broiler chickens infected with *S. pullorum*, this could provide a proliferative signal that alleviates spleen damage by promoting cell proliferation and inhibiting cell apoptosis.

P50, Rel A (also known as p65), and Rel B are the related transcription factors of the NF-κB family [47] that enable the activation of the target gene NF-κB expression and result from the regulation and function of an immune response [48]. Some studies have shown that NF-κB activity is induced during various physiological stress conditions [49, 50], resulting in the activation of the transcription factors P50, P65, and Rel B, ultimately leading to cell death. Here, we also found optimal activation of P50, P65, and Rel B in the spleen during *S. pullorum* infection. A previous study reported that *Salmonella* activated NF-κB, raising the possibility that *S. pullorum* stress may intensify the P50, P65, and RelB contributions to NF-κB activity [51]; the present results also verified the above specific candidates of recognition pattern receptors. The innate immune system expresses a wide variety of recognition pattern receptors and transcription factors that mediate pathogen recognition, leading to production of proinflammatory cytokines, pathogen uptake and destruction, antigen processing and presentation, and initiation of the adaptive immune responses, which causes NF-κB activation. NF-κB activation is followed by the degradation of the transcription factors P50, p65, and RelB. Therefore, we speculate that P50, P65, and RelB signal transduction was attenuated, which caused the NF-κB activation to be inhibited by the complex and, ultimately, the retrogression of the inflammatory response to spleen injury. As previously described, Gln can inhibit the expression of NF-κB to regulate the inflammatory response [52]. Our data confirmed that Gln can inhibit P50, P65, and RelB expression in the spleen, which is associated with the significant inhibition of NF-κB activation in spleen tissue. Finally, the effect of Gln on the transcription factors led to concomitant attenuation of the inflammatory cytokine expression in the spleen. These results may provide the first indication of a mechanistic explanation for the anti-inflammatory effects of Gln in the spleen.

## Conclusions

Taken together, our results indicated that *S. pullorum* affects the innate immune response by decreasing the spleen and bursa of fabricius relative weights, serum immunoglobulins concentrations, and the spleen MDA5 and LGP2 mRNA expression levels, and increasing the mRNA expression levels of the spleen NOD1, TLR2, TLR4, DAI, MAVS, P50, P65, and RelB of chickens, consequently increasing the susceptibility of the host to infection. However, dietary supplementation with Gln may benefit the cell-mediated host immune responses by activating the main pattern recognition receptor-dependent signaling pathways during *S. pullorum* infection. This important finding suggests the need for further evaluation of Gln supplementation to support innate as well as lymphocyte cell-mediated immunity in *S. pullorum*-infected broiler chickens.

## Data Availability

All data generated or analyzed during this study are included in this published article.
